# Recent advances in understanding and managing infantile colic

**DOI:** 10.12688/f1000research.14940.1

**Published:** 2018-09-07

**Authors:** Siel Daelemans, Linde Peeters, Bruno Hauser, Yvan Vandenplas

**Affiliations:** 1 KidZ Health Castle, UZ Brussel, Vrije Universiteit Brussel, Brussel, Belgium

**Keywords:** Infant, colic, crying, distress

## Abstract

A newborn brings joy to the family. Crying belongs to the spectrum of normal behaviour of young infants. However, although it occurs in about 20% of all infants, unsoothable and persistent crying in young infants distresses the family, although it is usually benign. The aetiology of infantile colic remains unknown, although an unbalanced gastro-intestinal microbiome, increased intestinal permeability, and chronic inflammation are involved, as well as behavioural factors, including over- and under-stimulation. It is a challenge for healthcare professionals to decide when organic disease needs to be excluded. Parental stress is a reason for babies to cry more, inducing a vicious cycle. Therefore, parental reassurance with explanatory guidance is the cornerstone of management. The placebo effect is estimated to be as high as 50%. If an intervention is felt to be necessary to offer further support to the baby and family, it is important to choose the options for which there is some efficacy without adverse effects. There is evidence that some specific probiotic strains such as *Lactobacillus reuteri* DSM 19378, especially in breastfed infants, are effective. However, there are also promising data for some synbiotics and/or killed or tyndallized bacteria, as well as substances decreasing intestinal permeability. Formula management with extensive and/or partial hydrolysates may also bring relief. But, above all, offering parental support remains imperative.

## Introduction

Infantile colic represents a major cause of discomfort and distress for the infant, caregivers, and even healthcare providers. Despite 50 years of research, its pathogenesis is not fully understood, and optimal management is debated. The aetiology of infant crying is multifactorial and related to feeding difficulties and disorders, dysmotility, hormone alterations, or behavioural factors. The concept of an aberrant gut microbiota in infants suffering from colic has been developed, suggesting its influence on gut motor function and gas production and emphasising a possible role for chronic inflammation. These new findings open up new perspectives in the management of infantile colic such as probiotic administration.

## What is infantile colic?

Infantile colic is classified as a functional gastro-intestinal disorder (FGID). FGIDs occur in about 50% of infants, and up to 75% of these infants present with symptoms of more than one FGID ^1, 2^. According to literature reviews, infantile colic is estimated to occur in about 20% of all infants ^1^.

Originally, Wessel and colleagues defined infantile colic in 1954 as episodes of crying for more than three hours a day for more than three days a week for three weeks in an otherwise healthy child ^3^. Recently, a group of experts published adapted definitions for both daily practice and clinical research ^4^. According to this definition, the diagnosis of colic for clinical purposes must include all of the following: i) an infant who is younger than 5 months of age, ii) presenting with recurrent prolonged periods of infant irritability, fussing, or crying reported by parents that occur without obvious cause and cannot be prevented or resolved by caregivers, iii) without evidence of infant failure to thrive, fever, or ill health. However, for clinical research purposes, in order to diagnose infant colic, the child must meet the clinical criteria plus both of the following: i) caregiver reports that the infant has cried or fussed for three or more hours/day during three or more days in seven days in a telephone or face-to-face screening interview with a researcher or clinician and ii) total 24-hour crying plus fussing in the selected group of infants is confirmed to be three hours or more when measured by a single, prospectively kept, 24-hour behaviour diary ^4^.

The crying occurs often in the evening ^5^. Colic is reported to occur equally frequently in breastfed and bottle-fed infants and in both sexes. Colic is much more frequent in the first 6 weeks (17–25%) compared with 11% at 8–9 weeks of age and 0.6% at 10–12 weeks of age ^6^. Therefore, many guidelines don’t recommend performing diagnostic procedures before the age of four to six months.

Infantile colic typically does not result in long-term problems, although the statement continues with “the crying can cause frustration for the parents, depression following delivery, excess visits to the doctor, and child abuse” ^5^. Colic definitely causes stress in the family and impairs family and infant quality of life. There are some indications that babies with colic become children at increased risk for recurrent abdominal pain or become adults who have more frequent FGIDs or mood disorders than a control healthy infant population without colic ^7^. It has been suggested that infants with infantile colic are at increased risk of developing migraine ^8^. However, well-designed long-term, prospective, observational studies are needed to endorse the few reports on possible associations between infantile colic and later health outcomes.

## Aetiology

The cause of colic is unknown. The diagnosis of infantile colic requires ruling out other possible causes ^5^. Alarm symptoms include fever, poor activity, or a swollen abdomen. But less than 5% of infants with excess crying have an underlying organic disease ^5^. One of the major diagnostic challenges is that crying is part of the symptom spectrum of many conditions that occur relatively frequently in infants such as gastro-oesophageal reflux, cow milk protein allergy, etc. However, crying as a “single” or “solitary” manifestation in one of these conditions is rare. In other words, many infants with troublesome gastro-oesophageal reflux do cry a lot, but crying as a single manifestation is a rare presentation of gastro-oesophageal reflux.

Infantile colic pathophysiology is poorly understood. Swallowed air has been suggested as a contributing factor; whether aerophagia should be considered as a cause or consequence is a matter of debate. Over- and under-stimulation are also recognised as causes of infant irritability. Some experts consider that infantile colic is due to gastro-intestinal discomfort or intestinal cramping ^9^. Colicky infants display gut microbiota dysbiosis, barrier alterations, and mild chronic gastro-intestinal inflammation ^5, 10, 11^. Faecal samples taken from infants suffering from colic led to visceral hyperalgesia in recipient mice, possibly as a result of microbiota dysbiosis ^12^. Visceral hypersensitivity could be an important aetiological factor involved in the prototypical colic crying behaviour. Similar perturbations are reported in irritable bowel syndrome. The gastro-intestinal colonisation may develop slower in colicky infants, with a lower diversity and stability ^13^. The microbiome of colicky infants has low levels of bifidobacteria and lactobacilli, including species with anti-inflammatory effects. There is a decreased number of butyrate-producing species ^14, 15^. *Escherichia coli* were reported to be more abundant in the faeces of infants with colic than in those of healthy infants. Proteobacteria, including species producing gas and inflammation, are increased ^13^. *Klebsiella* species are detected in larger amounts in colic than in control patients, while *enterobacter* and *pantoea* species are present only in the controls ^16^. The presence of chronic inflammation is illustrated by the fact that faecal calprotectin levels were two-fold higher in infants with colic than in control infants ^17^, although older reports contradict this finding and report similar calprotectin levels in infants with and without infantile colic ^18^.

In all children, during the first few months of life, intestinal mucosal immaturity implies an incomplete gut integrity, thus allowing the passage of large molecules into the blood ^19^. Breastfed and formula-fed infants with infantile colic have an increased transmission of the macromolecule human α-lactalbumin across the gut compared with healthy, age-matched infants ^19^. While the development of barrier function occurs *in utero*, there is ongoing postnatal maturation, and multiple factors can induce postnatal intestinal barrier maturation, including growth factors, hormones, nutrients, and microbes ^20^.

## Management

Many paediatricians do not feel very confident in dealing with infantile colic, which is related to the fact that there is no evidence-based approach to manage persistent infant crying ^21^. Parental reassurance is the cornerstone of the management of infantile colic ^5^. Since infantile colic decreases from the age of three months and disappears by the age of four to six months, it is obvious that any proposed therapeutic intervention should be devoid of any risk of adverse effects ^6^. The placebo effect of any therapeutic intervention is important and may reach up to 50% ^20^. However, some parents and families tolerate crying better than others. Interventions are targeted to decrease crying and bolster the infant–family relationship. Reassurance that the infant is not sick is of major importance, and it may necessitate multiple consultations before the parents become confident with this idea ^22^. The goals of management are to help the parents cope with the crying and to prevent long-term sequelae in the parent–child relationship. Infant colic is a risk factor for child abuse ^23^.

Although there is no evidence for the benefit of soothing techniques, they cost nothing, are devoid of adverse effects, and have the advantage that active recommendations are given to the parents. There are the five manoeuvres (the five S’s), a group of reflexes (vestibular, auditory, and tactile) that work together to calm the baby: swaddling, shushing, stomach position, swinging, and sucking. Regarding manipulation therapies, a Cochrane analysis concluded that studies were generally small and methodologically prone to bias ^24^. Although the majority of the included trials appeared to indicate that the parents of infants receiving manipulative therapies reported fewer hours of crying per day than parents whose infants did not, based on contemporaneous crying diaries, most studies were subject to a high risk of performance bias because those carrying out the assessment (the parents) were aware of whether or not they were participating in the intervention group ^24^. Osteopathy is considered to have no benefit, and this is also based on the fact that there are hardly any data ^25^.

Infants with colic seem to be in pain. However, the evidence of the effectiveness of pain-relieving agents for the treatment of infantile colic is sparse and prone to bias ^26^. Moreover, similar to irritable bowel syndrome in adults, infantile colic may rather be the consequence of a decreased pain threshold than an increased intensity of pain, as the microbiome is associated with the level of pain threshold. Benefits, when reported, were inconsistent ^26^.

Despite some positive reports from parents ^27^, there is no evidence to support the use of simethicone as a pain-relieving agent for infantile colic ^26, 28– 30^. Simethicone is an anti-foaming agent used to reduce bloating, discomfort, or pain caused by excessive gas.

In breastfed infants, the evidence for maternal dietary manipulation is weak ^25, 31^. There is no evidence to recommend an elimination diet, e.g. without cow’s milk, for a breastfeeding mother to improve infant crying. Two systematic reviews from 2012 of small randomised trials with methodologic limitations suggest that extensive hydrolysate formulae may reduce distress in infants with colic ^28, 32^. Fibre-supplemented formulae had no effect ^32^. If the baby is growing well and if crying is the only symptom, there is no evidence that an extensive hydrolysate will be of help ^25^. In atopic families, there may be some benefit, although this is advised based on clinical thinking and experience and not on evidence ^25^. Breastfeeding should be continued.

Limited data suggest that using a partially hydrolysed infant formula may be of some benefit in reducing infantile colic in cases where cow’s milk allergy is not suspected in formula-fed infants ^20^. Whether this is due to the protein hydrolysate or to the reduced lactose content of these formulae is not clear ^33^. Some old data suggest a beneficial effect of lactose reduction in the infant’s diet as a result of administration of exogenous lactase, based on the theory that infants with colic may have transient lactose intolerance because of slow maturation of lactase ^34, 35^. There is some evidence that oral lactase can reduce crying time ^34^. The United Kingdom National Institute for Health and Care Excellence (NICE) guidelines recommend a trial of 2 weeks’ administration of lactase. In some of these studies, the tested formula contains another lipid and/or prebiotics or probiotics as well. Thus, although dietary changes can result in a beneficial outcome, there is limited evidence as to which specific dietary change is causing the effect ^20^. Removal of poorly digested carbohydrates from the infant’s diet has promise, but additional clinical studies must be conducted before a recommendation can be made ^32^. In breastfed infants, the evidence for maternal dietary manipulation with lactase, sucrose, or glucose is weak ^25, 31^.

Dietary advice can best be summarised as follows. For breastfed infants, a monitored low-allergen maternal diet avoiding cow’s milk and dairy food with appropriate intake of vitamins and minerals should be adopted for at least two weeks. If there is no improvement, the elimination diet should be stopped. Infantile colic is not a reason to stop breastfeeding. For bottle-fed infants, the first-line approach recommended in infants who are not suspected of suffering from cow’s milk protein allergy are formulae with partially hydrolysed whey proteins, with reduced lactose with prebiotic oligosaccharides and probiotics. It is important to recommend only the formulae for which there is some evidence of efficacy in the literature. Extensively hydrolysed formulae based on casein or whey could be useful in children with cow’s milk protein allergy; most of the time, these infants present with more severe colic and with other manifestations of atopic disease, such as atopic dermatitis. It is crucial that dietary changes are performed under the supervision of a healthcare provider ^36^.

Available evidence shows that herbal agents, sugar, and cimetropium bromide cannot be recommended for infants with colic ^26^. Preparations containing fennel are suggested to be effective in breastfed infants, with an overall mean difference of –72.1 minutes of crying/day ^31^. Mentha piperita was reported to be beneficial in one trial ^37^. Dicycloverine, also known as dicyclomine, relieves muscle spasms in the gastro-intestinal tract through an apparent mechanism of nonselective smooth muscle relaxation, and that presents a range of anticholinergic side effects such as a dry mouth, nausea, and, at higher doses, deliriant effects. Since efficacy was never shown, and because of the adverse effects, this drug should not be used in infantile colic. Cimetropium bromide is a belladonna derivative. Evidence does not support its use in infantile colic ^26, 28^. Reported adverse effects include constipation, pupil dilatation with loss of accommodation, photophobia, reduced bronchial secretions, heart rhythm variability, and skin flushing.

Acid blockers such as proton pump inhibitors, which are the recommended treatment for gastro-oesophageal reflux disease, do not provide any relief in colicky infants ^38^. Although crying is frequently reported in infants with gastro-oesophageal reflux disease, crying itself is not an indicator of the condition. Moreover, acid-blocking medication contributes to the development of an unbalanced gastro-intestinal microbiome, which is a risk factor for allergy and gastro-intestinal and respiratory tract infections ^39^.

What about probiotics? *Lactobacillus reuteri* DSM 17938 is a well-documented, effective, and safe treatment mainly in breastfed infants ^30, 40– 43^. Analysis of response rates showed that infants receiving *L. reuteri* DSM 17938 had a 2.3-fold greater chance of having a 50% or greater decrease in crying/fussing time compared to controls ^40^. However, two trials in mainly formula-fed infants failed to show a significant benefit ^44, 45^.

The reasons for the crying in formula-fed infants may differ from those in breastfed infants. In a recent trial, mechanisms of action have been suggested ^46^. Breastfed infants with colic treated with *L. reuteri* DSM 17938 for 30 days had a significantly decreased crying time and an increased FOXP3 concentration, resulting in a decreased RORγ–FOXP3 ratio ^45^. The treatment reduced faecal calprotectin ^46^. The outcome of trials with *Lactobacillus rhamnosus* GG was negative ^47, 48^. These findings suggest once more that data obtained with one probiotic strain cannot be extrapolated to other strains. There is one trial suggesting efficacy of a synbiotic (fructo-oligosaccharides, *Lactobacillus casei*, *L. rhamnosus*, *Lactobacillus acidophilus*, *Lactobacillus bulgaricus*, *Streptococcus thermophilus*, *Bifidobacterium breve*, and *Bifidobacterium infantis*) ^49^. A partial whey hydrolysate with reduced lactose, *Bifidobacterium lactis* BB12, and galacto-oligosaccharides was shown to be effective in infantile colic ^50^. In all trials, probiotic supplementation was not associated with any adverse events. Although probiotics are Generally Regarded As Safe (“GRAS” status assigned by the US Food and Drug Administration), case reports of mucormycosis and probiotic-associated septicaemia have been reported.

It is not clear to what extent the microorganisms administrated need to be living. Although, as said above, no safety issue has been reported, it is obvious that conservation of the product would be much easier if survival is not important. According to some *in vitro* data, beneficial gastro-intestinal effects may also be achieved with dead microbes, which produce a similar immunomodulatory effect and anti-inflammatory response within the gastro-intestinal mucosa to those produced by live microbes ^51, 52^.

Although increased permeability is one of the hypothesised aetiologies of colic, few intervention trials focused on this aspect. APT198K or Aprotecol® contains Xyloglucan 0.6 g/10 ml, which forms a mechanical barrier over the wall of the gastro-intestinal tract, and tyndallized probiotics have been tested in some trials. Tyndallization is a method of sterilisation developed by physicist Dr John Tyndall during the 19th century. Tyndallization essentially consists of heating the substance to boiling point (or just a little below boiling point) and holding it there for 15 minutes, three days in succession. The resting periods between heating periods allow surviving spores to develop into bacterial cells, which will then be destroyed by the next day’s heating. Tyndallized probiotics *L. reuteri* SGL01 100 × 10 ^9^ colony-forming units (CFU)/g and *B. breve* SGB01 100 × 10 ^9^ CFU/g were recently compared to lactase in a clinical trial ^53^. Both interventions reduced crying time, which may be related to the placebo effect or to the fact that both interventions are indeed effective, but APT198K decreased the mean duration per crying episode significantly more than a lactase dietary supplement in infants with colic ^53^. A trial comparing a combination of *Matricaria chamomilla*, *Melissa officinalis*, and tyndallized *L. acidophilus* (HA122) with *L. reuteri* DSM 17938 and with simethicone showed that simethicone was not effective but both other arms were equally effective ^29^. At high-dose and in rats, simethicone has also been shown to decrease intestinal permeability ^54^.

One should be very careful in recommending or even accepting so-called alternative treatments because, besides the fact that they are unlikely to be effective, they may be harmful. Homeopathic remedies often are considered nontoxic because of the low concentrations of active ingredients. However, the labels of homeopathic products may not report all of the ingredients, some of which potentially may be toxic ^55^. As an example, gas chromatography-mass spectrometry analysis of a homeopathic remedy for colic that was associated with an increased risk of apparent life-threatening events found that it contained ethanol, propanol, and pentanol in addition to three potentially toxic substances that were listed as active ingredients ( *Citrullus colocynthis* [bitter apple], *Veratrum album* [white hellebore], and *Strychnos nux-vomica* [strychnine tree]) ^56^. Spinal manipulation does not reduce the duration of crying associated with colic and does not enhance recovery ^25^. However, at least two deaths due to spinal manipulation were reported ^57^.

## Prevention

Prevention is, of course, always preferable to management, especially in the absence of an effective treatment. *L. reuteri* DSM 17938 was shown in a preventive trial to decrease all FGIDs, including colic ^58^. In another prevention trial, the same strain was shown to result in a lower number of paediatric consultations related to episodes of infant colic than the control group ^59^. *L. reuteri* DSM 17938 supplementation could reduce parental discomfort due to infantile colic ^59^. The combination of formula fermented with *B. breve* and *S. thermophilus* (Lactofidus) in combination with prebiotic short-chain galacto-oligosaccharides and long-chain fructo-oligosaccharides resulted in a lower overall crying time and a lower incidence of infantile colic in healthy term infants ^60^. These data suggest that killed bacteria might be effective as well. The symptoms of regurgitation, constipation, and infant crying and colic were decreased with a formula containing intact protein and synbiotics (fructo-oligosaccharides and *B. lactis*) ^61^.

## Conclusion

Infantile colic is distressing to parents whose infant is inconsolable during crying episodes. The physician’s role is to make sure that the crying is not a result of other causes, offer balanced advice on treatments, and provide support to the family. Above all, parents need reassurance that their baby is healthy and that colic is self-limited with no long-term adverse effects ^62^.

Treatment possibilities for infantile colic are limited. Evidence suggests that manipulation of the gastro-intestinal microbiome may be of some benefit. *L. reuteri* DSM 17938 is the best-studied probiotic for colic, especially in breastfed infants. Some data suggest that dead or tyndallized bacteria may be of benefit as well, perhaps as a result of reduced gut permeability. A Cochrane review showed that the evidence for the effectiveness of pain-relieving agents for the treatment of infantile colic is scanty and prone to bias ^26^. Data on pain-relieving agents for infantile colic are from old studies with a small number of patients with serious methodological limitations. As a consequence, additional randomised controlled trials are needed.
